# Cardiac Resynchronization Therapy beyond Nominal Settings: An IEGM-Based Approach for Paced and Sensed Atrioventricular Delay Offset Optimization in Daily Clinical Practice

**DOI:** 10.3390/jcm12124138

**Published:** 2023-06-20

**Authors:** Mariëlle Kloosterman, Fenna Daniëls, Eva Roseboom, Michiel Rienstra, Alexander H. Maass

**Affiliations:** 1 University Medical Center Groningen, Department of Cardiology, University of Groningen, 9712 CP Groningen, The Netherlands; m.kloosterman@umcg.nl (M.K.); f.daniels@umcg.nl (F.D.); e.roseboom@umcg.nl (E.R.); m.rienstra@umcg.nl (M.R.); 2 Department of Cardiology, Isala Hospital, 8025 AB Zwolle, The Netherlands

**Keywords:** optimization, heart failure, AV delay, intracardiac electrogram, echocardiography

## Abstract

Optimization of the atrioventricular (AV) delay has been performed in several landmark trials in cardiac resynchronization therapy (CRT), although it is often not performed in daily practice. Our aim was to study optimal AV delays and investigate a simple intracardiac electrogram (IEGM)-based optimization approach. 328 CRT patients with paired IEGM and echocardiography optimization data were included in our single-center observational study. Sensed (sAV) and paced (pAV) AV delays were optimized using an iterative echocardiography method. The offset between sAV and pAV delays was calculated using the IEGM method. The mean age of the patients was 69 ± 12 years; 64% were men, 48% had ischemic etiology of heart failure. During echocardiographic optimization, an offset of 73 ± 18 ms was found, differing from nominal AV settings (*p* < 0.001). Based on the IEGM method, the optimal offset was 75 ± 25 ms. The echocardiographic and IEGM-generated AV offset delays showed good correlation (R^2^ = 0.62, *p* < 0.001) and good agreement according to Bland-Altman plot analysis. CRT responders had a near zero offset difference between IEGM and echo optimization (−0.2 ± 17 ms), while non-responders had an offset difference of 6 ± 17 ms, *p* = 0.006. In conclusion, optimal AV delays are patient-specific and differ from nominal settings. pAV delay can easily be calculated from IEGM after sAV delay optimization.

## 1. Introduction

Cardiac resynchronization therapy (CRT) is a cornerstone in the management of patients with chronic systolic heart failure and conduction delays [1,2]. Landmark trials have demonstrated improvements in symptoms and cardiac function and reductions in morbidity and mortality in selected patients who are refractory to optimal medical therapy and fulfill the criteria of QRS > 130 ms and left bundle branch block (LBBB) QRS morphology (or QRS > 150 ms and non-LBBB QRS morphology), ejection fraction < 35%, and New York Heart Association (NYHA) class II, III, or IV [3,4,5,6,7]. Despite stringent patient selection to increase clinical response rates, many fail to show clinical improvement. One way to potentially improve clinical outcomes is increasing biventricular pacing rates, for example, by adequate treatment of atrial tachyarrhythmias and premature ventricular complexes. Another is device optimization by including individualized programming of the atrioventricular (AV) delay [8,9].

While individual optimization of the AV delay has been performed in several landmark CRT trials, in daily clinical practice, optimization of the AV delay is often not performed, mostly due to the time-consuming nature of the different echocardiographic techniques [10]. Furthermore, despite improvements in left ventricular diastolic and systolic function in the acute setting after optimization, [11,12,13,14] a neutral effect on clinical and echocardiographic outcomes was found in several randomized and nonrandomized studies [15,16].

The multitude of AV optimization techniques and the absence of a gold standard on AV device optimization reflects the current European Society of Cardiology (ESC) Guidelines that refrain from a recommendation on AV optimization but mention that AV optimization may be considered for patients who have had a disappointing response to CRT, [1,2]. Additionally, it remains difficult to identify those that may benefit. Even in a published practical guide to avoiding non-responders to CRT, no consensus on the preferred AV optimization technique and timing was reached, albeit that systematic routine optimization in all CRT recipients was suggested not needed until less time-consuming techniques have been investigated further [9]. Additionally, there is little evidence on optimal paced AV delays, and whether atrial pacing is beneficial in CRT patients remains controversial [15,17,18,19,20,21]. Since right atrial pacing, with its resultant delay of left atrial contraction, seems associated with a lower degree of left ventricular (LV) resynchronization and a reduction in LV filling times, AV delay optimization might even be more important in these patients [19].

Nevertheless, it seems important for physicians who manage CRT patients in daily clinical practice to be familiar with optimization strategies, and have a simple method readily available, since it is unlikely that nominal, out-of-the-box settings are the “one size fits all” solution for all patients treated with CRT. The aim of the present study was to analyze and compare optimal AV delays determined by the iterative method to nominal settings and propose a new, simple intracardiac electrogram (IEGM)-based optimization approach to determine optimal offset for atrial pacing in patients undergoing CRT optimization in a real-world clinical setting.

## 2. Materials and Methods

### 2.1. Patient Population

This was a single-center, observational study performed at the University Medical Center Groningen in the Netherlands. Consecutive patients without permanent atrial fibrillation who received a CRT device from March 2010 until November 2016 underwent routine AV optimization. Our CRT implantation protocol has been described before [21]. Eligibility criteria for CRT implantation were based on the recommendations in the ESC guidelines at the time of implantation [22]. At the time of this study, written informed consent was not necessary for anonymously handled data that were gathered during routine patient care.

### 2.2. Device Implantation

Patients were implanted with a CRT defibrillator or pacemaker. Devices were used from Abbott (Abbott Park, IL, USA), Biotronik (Berlin, Germany), Boston Scientific (Marlborough, MA, USA), and Medtronic (Minneapolis, MN, USA). Right atrial and right ventricular leads were implanted via transvenous access. The right atrial pacing lead was fixated in the right atrial appendage if present and accessible; otherwise, the lead was fixated to the anterior or lateral free wall. The right ventricle lead was placed in an apical position. The LV lead was placed transvenously via the coronary sinus; location depended on venous vasculature. In case of an inability to achieve adequate LV lead positioning, epicardial leads were placed via video-assisted thoracoscopic surgery as previously described [23].

### 2.3. Echocardiographic Optimization Protocol

Patients received echocardiography-based AV delay optimization approximately one month (interquartile range 28–43 days) after implantation. Echocardiographic images were obtained using a standardized protocol, and the iterative method was used to optimize AV delays. The iterative method studies transmitral flow and evaluates ventricular filling. By screening for A-wave truncation, which requires prolonging the AV interval, or fusion of E and A waves, which indicates too short diastolic filling and necessitates AV interval shortening, AV times were optimized. In short, a long AV interval was programmed, which was subsequently gradually shortened to 20-ms steps. The decrement of AV delay was continued until truncation of the A wave was observed on mitral inflow Doppler. Then the AV interval was gradually prolonged in 10-ms steps until maximal E and A wave separation was obtained and mitral valve closure coincided with or occurred shortly after, the atrial contraction. This was performed both during intrinsic sinus rhythm (sensed AV delay) and during atrial pacing (paced AV delay) at a heart rate just above intrinsic sinus rhythm (see Figure S2 online for a depiction of the iterative method during intrinsic sinus rhythm and atrial pacing). All optimizations were performed during resting conditions in a supine position by one experienced cardiologist (AHM).

### 2.4. IEGM Method

**Figure 1:**
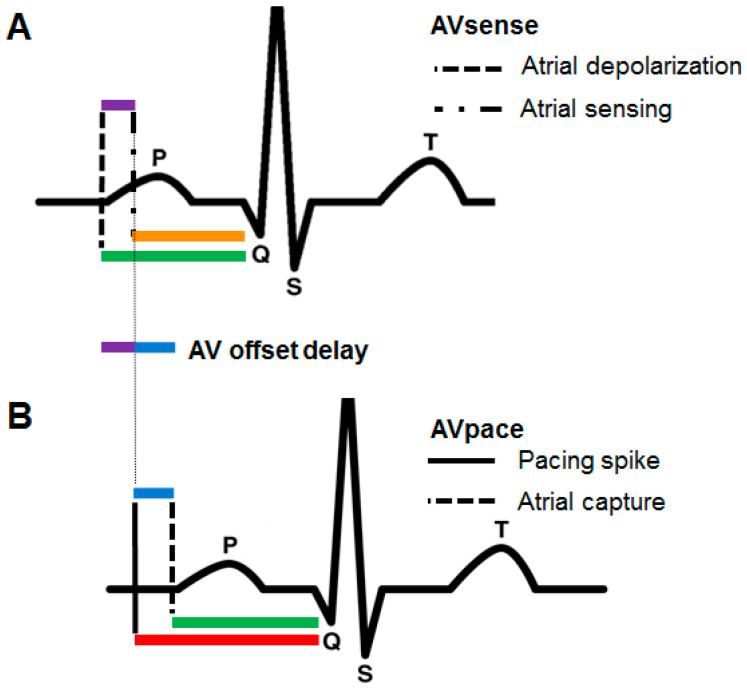
Difference between sensed and paced AV delays. (**A**) When a sensed P wave occurs, the effective PQ interval (green line) is longer than the sensed AV delay (orange line) because of the latency in atrial sensing (purple line). (**B**) When a paced P wave occurs, the effective PQ interval (green line) is shorter than the programmed AV delay (red line) because of the latency in atrial capture (blue line). The latency in atrial sensing and atrial capture causes the AV offset delay (blue and purple lines combined).

During AV optimization, optimal AV delays have to be measured during atrial sensing and atrial pacing. The measured AV interval during atrial sensing starts when the atrial depolarization is detected by the CRT and commonly occurs 20 to 60 milliseconds after the onset of the P wave seen on a surface ECG. Conversely, the measured AV delay initiated with atrial pacing commences immediately with the pacing artifact, not with atrial depolarization. Therefore, the AV delay that follows a sensed atrial event should be shorter than one that follows a paced atrial event in order to obtain similar functional AV delays (Figure 1).

**Figure 2:**
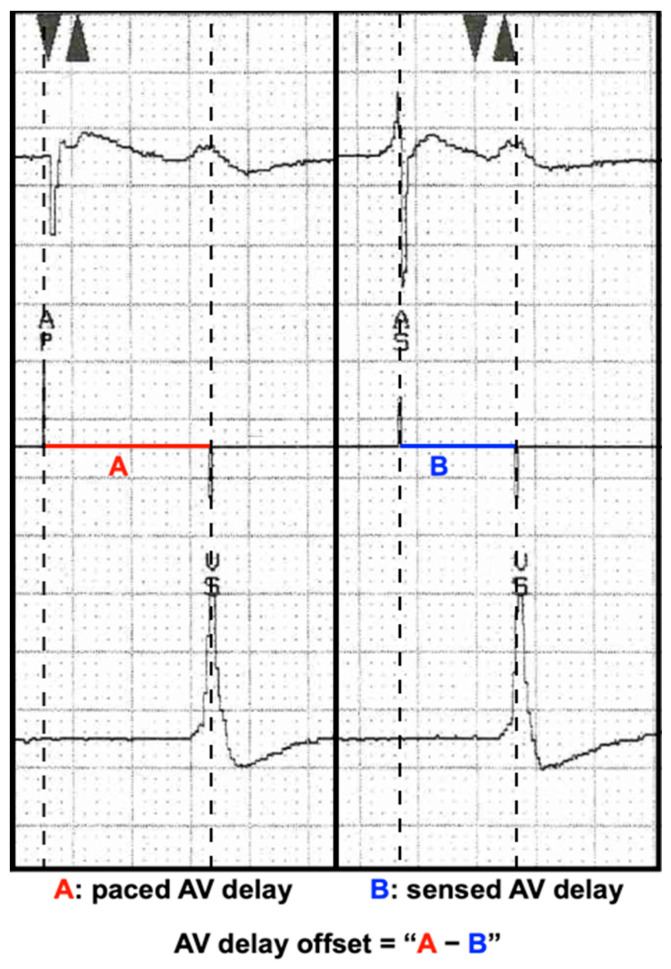
How to calculate the IEGM-based AV offset delay. Line A shows the paced AV delay on the IEGM, which is the time between the atrial pacing spike and the first depolarization of the ventricle. Line B represents the sensed AV delay on the IEGM, which is the time between the onset of atrial depolarization and the first depolarization of the ventricle. The IEGM-based AV offset delay is the difference between line A and line B.

During echocardiographic optimization, two IEGM transmissions were printed, one during intrinsic sinus rhythm and one during atrial pacing at a rate slightly above sinus rhythm. Provided information on the IEGM differs between CRT manufacturers (See Figure S3 online), but in general, during atrial sensing and—pacing, the AV delay was calculated by measuring the time difference between the P wave or pacing spike, respectively, and the first ventricular depolarization. Subtracting these values gave the IEGM-based AV delay offset (Figure 2). Offsets were calculated afterward using IEGMs without knowledge of the optimal delays during echocardiographic optimization and blinded for patient specifics.

### 2.5. Follow Up

After six months, patients underwent echocardiography to study response to CRT. The response was defined as an increase in LV ejection fraction (LVEF) of ≥10%.

### 2.6. Statistical Analyses

Patient demographics and characteristics are presented for the study population. Continuous variables are expressed as mean with standard deviation (SD). Categorical variables are summarized as frequencies and percentages. The difference in heart rate during sensed- and paced AV optimization was determined using a paired-sample t-test. The Bland-Altman plot method was used to test whether the offset values given by the two techniques were congruent. Pearson’s correlation coefficient was used to study the degree of correlation between the IEGM and echocardiography-determined offset. Data analysis was performed with SPSS 23.0 for Windows (SPSS Inc., Chicago, IL, USA). A p-value of ≤0.05 was considered significant.

## 3. Results

A total of 417 patients were included. In 328, paired IEGM and echocardiography optimization data were available, forming the final study population. Reasons for an incomplete dataset were sinus arrest (n = 7), atrioventricular block (n = 63), or supraventricular arrhythmias (n = 19) preventing paired data collection (see Figure S1 online for flow chart).

The mean age was 69 ± 12 years; 64% of patients were men, 48% had ischemic heart disease, and the mean ejection fraction was 25 ± 8%. In 298 patients (91%), the left ventricular lead was positioned in a tributary of the coronary sinus overlying the LV free wall. In total, 30 (9%) patients in whom no transvenous LV lead could be placed received an epicardial LV lead. The clinical characteristics of the patients are shown in Table 1.

### 3.1. Echocardiographic AV Optimization

**Figure 3:**
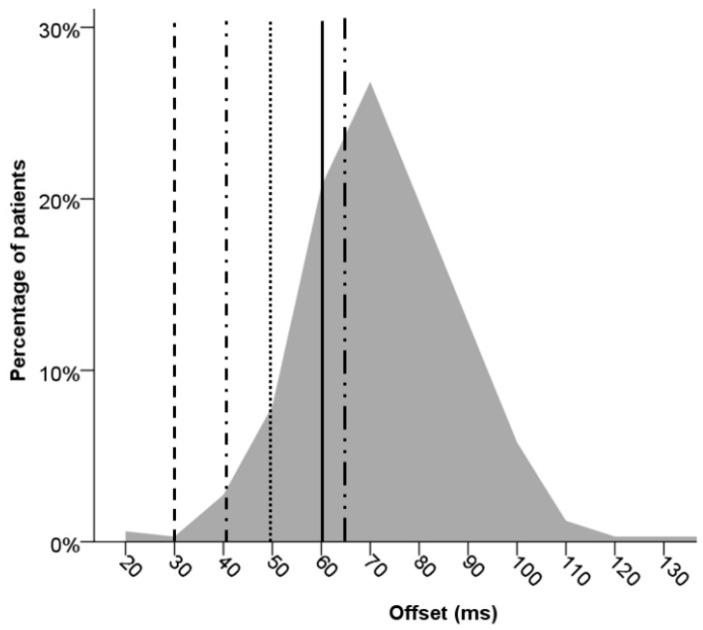
Sensed- and paced AV delays in the patient cohort compared to nominal device settings. Optimal atrioventricular (AV) delay offset is plotted for the total study population. Lines represent nominal programmed offset delays of five major manufacturers. A large percentage of patients (>67%) had AV offset delays that exceeded nominal settings.

Heart rate during sensed and paced echocardiographic AV optimization was 70 ± 12 bpm and 74 ± 12 bpm, respectively (*p* < 0.001). The mean optimal sensed AV delay was 75 ± 26 ms, mean optimal paced AV delay was 148 ± 32 ms, leading to an offset of 73 ± 18 ms (Table 2). The values differ from the nominal AV settings of five major manufacturers (Table 3, *p* < 0.001). As illustrated in Figure 3, 67.7% of patients had an offset that exceeded nominal settings.

### 3.2. IEGM-Based AV Delays

**Figure 4:**
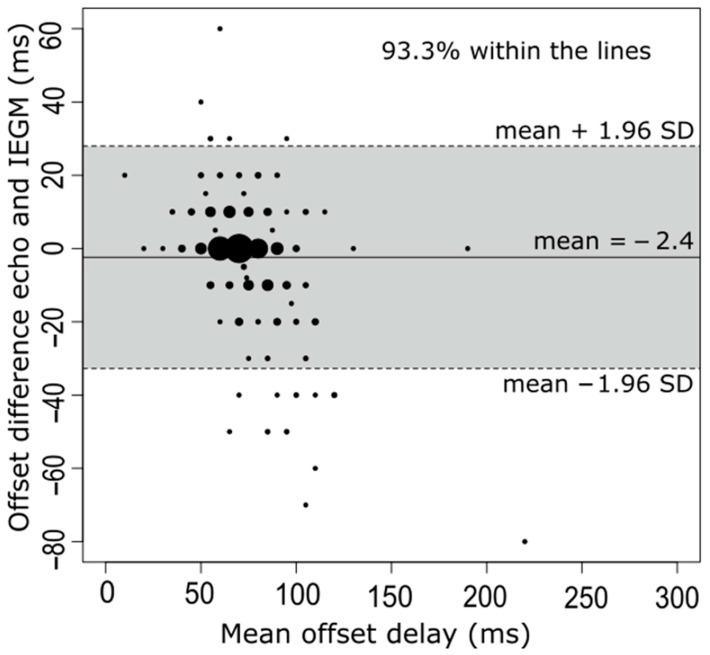
Bland-Altman plot of the difference between the two methods. Bland-Altman plot of differences in optimal AV offset delay measured by echocardiography and by IEGM. Lines and colored areas are indicative of upper and lower limits of agreement (95% confidence interval) and indicate margins of difference between the two techniques (0 ± 30 ms). The size of the dots represents the number of patients. AV, atrioventricular; IEGM, intracardiac electrogram; SD, standard deviation.

**Figure 5:**
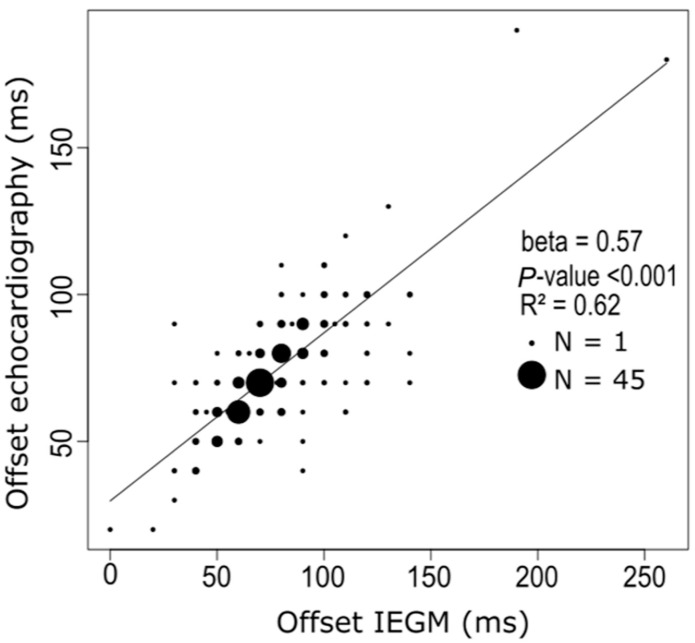
Correlation between the echocardiography and IEGM determined AV offset delay. The optimal offset between sensed and paced AV delays, obtained from the AV optimization and IEGM calculations, show a good correlation (R^2^ = 0.62, *p* < 0.001). The size of the dots represents the number of patients. AV, atrioventricular; IEGM, intracardiac electrogram.

Intrinsic sensed—and paced AV delays using the IEGM method were 194 ± 50 ms and 269 ± 59 ms, respectively. This led to an AV offset delay of 75 ± 25 ms based on IEGM values (Table 2). Using a Bland-Altman plot analysis in any single patient, the clinically acceptable correlation between echo- and IEGM-generated AV offset delays showed good agreement. In 93.3% of patients, differences in mean variations were within ± 2SD of the mean (Figure 4). Furthermore, the optimal offset between sensed and paced AV delays, obtained from the AV optimization and IEGM calculations, showed a good correlation (R^2^ = 0.62, *p* < 0.001) (Figure 5).

### 3.3. CRT Response

After six months, 71% of the study population was a CRT responder. The offset difference for IEGM and echo optimization was near zero for the responders (−0.2 ± 14 ms), while it was significantly longer for non-responders (−6 ms ± 17 ms, *p* = 0.006).

## 4. Discussion

Optimal AV delays are patient-specific and differ from nominal device settings. The main finding of this paper is that echo-derived and IEGM-generated offset delays show a good correlation. Identical echo and IEGM offsets were associated with CRT response. Therefore, in daily clinical practice, the IEGM approach may be used to calculate AV offset delay so that the optimal paced AV delay offset can be easily programmed after echocardiographic determination of optimal sensed AV delay.

### 4.1. AV Optimization Methods

Most large CRT trials applied some form of AV delay optimization, and it is unknown whether the beneficial effects of CRT in these trials would also be present without AV delay optimization. However, in daily clinical practice, nominal settings seem standard of care since current optimization techniques have several limitations, including their time- and resource-consuming nature, the limited reproducibility, and the intra- and inter-operator variability. Our results show that optimal AV delay in CRT patients exhibits great variability from patient to patient, suggesting that an empirically programmed AV delay interval is suboptimal in at least a subset of patients. In our study, 67.7% of patients had offset delays that exceeded nominal settings.

In clinical practice, there are many techniques for optimizing AV delays. Echocardiography has been considered the “gold” standard for optimization, given its relative ease and widespread availability. Echocardiography techniques include those that focus on mitral inflow (the Ritter method, the most commonly used iterative method, and the “fast- and simple” approach) or on the aortic pulsed-wave Doppler velocity time integral. Alternative techniques to optimize AV delay consist of impedance cardiography, finger photoplethysmography, hemodynamic optimization using noninvasive blood pressure measurements, acoustic cardiography, or peak endocardial acceleration [24,25,26]. Moreover, a flow pattern in the LV outflow tract in which there is coupling between mitral-aortic flow reversal and ejection flow has also been described as a way for determining optimal AV delay [27]. Additionally, there are multiple IEGM-based algorithms available, including QuickOpt (Abbott^®^), SmartDelay (Boston Scientific^®^), Adaptive-CRT (Medtronic^®^), and SonR (Microport^®^). These device-based algorithms use predefined formulas to identify the best combination of AV and VV timing. The Frequent Optimization Study using the QuickOpt Method (FREEDOM) trial compared IEGM-based AV optimization to conventional management (empiric programming or one-time non--IEGM-based optimization) in a randomized 1:1 design. The SMART-AV study compared the use of the SmartDelay AV-optimization algorithm versus echocardiography-optimized AV delay versus nominal settings in a randomized 1:1:1 design [15]. Both algorithms were safe and easy to use but were, in terms of efficacy, amount of reverse remodeling, and improvement in functional status, not better than routine clinical practice, which included echocardiography-guided optimization or nominal settings. In the ADAPTIV-CRT trial, the Adaptive-CRT algorithm, which continuously adapts delivery mode and timing settings, was non-inferior compared to echocardiography optimization methods [16]. Moreover, the SonR algorithm is a dynamic AV-optimization algorithm and weekly optimizes AV delays depending on heart rate and LV dP/dt. In the double-blind, randomized controlled non-inferiority Respond-CRT trial, the SonR technology was compared to weekly echo-guided optimization using the iterative method and was shown to be non-inferior to the AV and VV echo-guided approach, although the clinical response was in favor of the SonR sensor, especially in a specific patient population with atrial fibrillation or renal dysfunction [28]. Although the previously mentioned algorithms are less time-consuming than the echocardiographic optimization, a beneficial effect on clinical outcomes compared with the echocardiographic method has yet to be shown. Moreover, since these algorithms use predefined formulas, patient-specific echocardiographic or hemodynamic measurements are not taken into account. Our IEGM-based method to calculate optimal AV delay offset showed a good correlation with the echocardiographic method. Moreover, it is a combination of patient-tailored and time-efficient AV delay optimization, and the same method can be used for different manufacturers.

### 4.2. AV Delay: Selected Groups and Outcome Markers

Despite the neutral randomized studies in optimizing AV delays, experimental physiological and pathophysiological research supports the rationale to optimize AV delays [11,12,13,14,27]. Adequate patient selection, as well as the best method for optimizing, measuring, and assessing the effects of AV optimization, remains to be defined to establish clinical benefit.

The benefit of AV optimization may not lie in the conversion of non-responders to responders but in increasing the magnitude of CRT response. The MARC study has shown that reverse remodeling can be predicted by simple parameters, and the maximal effect may only be achieved in selected patients when adding AV optimization [29]. This might be especially true in women and patients receiving atrial pacing [15,30]. Moreover, baseline interventricular electrical dyssynchrony is associated with the reverse remodeling with CRT. Gold et al. showed an incremental benefit of AV delay optimization in patients with the longest interventricular delay, thus presumably improving the magnitude of CRT response in patients with long baseline interventricular delay [31]. Additionally, AV delay optimization has been associated with a higher percentage of biventricular pacing at follow-up and thereby improving hemodynamic parameters [32].

Current knowledge suggests that right atrial pacing, which introduces a prolongation of interatrial conduction leading to delayed left atrial systole, thereby curtailing ventricular filling, is associated with an increase in AF incidence and heart failure rehospitalization after CRT implantation [17,33]. Moreover, right atrial pacing seems associated with a lower degree of LV resynchronization and a reduction in LV filling times compared to VDD mode, thereby possibly abolishing some of the beneficial effects of CRT [18]. Moreover, a high burden of atrial pacing is independently associated with less symptomatic improvement and less LV reverse remodeling [33]. It is clear that the timing of ventricular pacing relative to right atrial paced events is significantly different compared to atrial sensing mode and needs to be delayed accordingly to achieve a comparable situation to intrinsic atrial activation. A study by Gold et al. has shown that too short AV delays negate a possible hemodynamic benefit of atrial pacing. They demonstrated that atrial pacing increased LV dP/dt_max_ and that hemodynamic response was linearly related to heart rate. To achieve a maximal increase in LV dP/dt_max_ during atrial pacing, a patient-specific increased AV delay was needed. In accordance with Bernheim et al., there was a trade-off between LV dP/dt improvement and reduced LV filling and ejection at higher rates [19]. Additionally, post hoc analyses in the SMART-AV delay trial found a trend towards greater reductions in LV end-systolic volume in patients with >30% atrial pacing and optimized AV delays compared to fixed AV delays [15]. These studies suggest that if programmed poorly, atrial pacing has the potential to curtail the beneficial effects of CRT. On the other hand, Martens et al. performed echocardiographic AV optimization and still showed the previously mentioned negative effects of RA pacing in CRT patients, which presumably could be even worse without AV optimization [33]. This highlights the importance of optimal-paced and sensed AV delays, especially in the subset of patients who require frequent atrial pacing. Moreover, AV delays are not static values and may vary in time, which could have attributed to the inconsistent long-term effects on clinical outcomes. Recurrent AV optimization could be of added value, although an optimal frequency has not been established yet [34,35]. Our described method offers a simple and fast way of AV optimization that may increase practice efficiency, simplify patient management, and ensure that paced AV delays are optimized. Additionally, our described method is suitable for periodic AV optimization.

Although AV delays may not increase CRT response in populations, individual patients may benefit from improved diastolic filling and reduction diastolic mitral valve insufficiency. The most optimal outcome marker of AV optimization might therefore be diastolic function and reduction in left atrial volumes [36]. If, after CRT implantation, ventricular reverse remodeling is absent during follow-up, AV optimization can result in atrial reverse remodeling. We have previously shown that in a subset of patients, AV optimization results in a significant improvement in LV diastolic filling time and filling fraction, even in the absence of ventricular reverse remodeling. In addition, the velocity time integral of transmitral flow improved, indicating not only longer but also augmented diastolic filling [36]. Importantly, atrial reverse remodeling is associated with an improved outcome in CRT patients [36,37].

### 4.3. Study Limitations

This study was relatively small and observational in design and had all the limitations inherent to this type of design. Data on the exact location of the right atrial lead and the amount of right atrial pacing are missing; therefore, its influence on AV delay could not be studied. CRT response could only be determined by an increase in LVEF because LVESV measurements were not systematically recorded at follow-up, leading to too few patients with paired LVESV measurements to determine LV reverse remodeling. One important limitation of the IEGM method is that it is a method to identify the optimal offset between sensed and paced AV intervals and not the absolute value of each AV interval. This means the optimal sensed AV interval should be determined, for example, by the echocardiographic method or by integrated device algorithms. From all available optimization methods, we compared the IEGM method only to the echocardiographic iterative method. However, the iterative method is the most commonly used echocardiographic optimization technique, is relatively easy, and was, among others, used in the CARE-HF and SMART-AV trials. In both the IEGM and the iterative method, we did not correct the difference in AV delay with different RR intervals. This could have resulted in a higher difference in offsets compared with nominal AV settings. Moreover, the need to adapt AV delays to physiological alterations during exercise and during follow-up if anatomical reverse remodeling takes place was not studied and warrants further investigation. Additionally, our IEGM method should be investigated at higher heart rates and might be an easy option to determine heart rate-dependent offsets. Additional multicenter studies are needed to explore the relative merits of AV optimization, hereby also focusing on atrial reverse remodeling and -function in order to redefine the role of AV optimization and the use of the described IEGM method. Moreover, this should be compared to the integrated device algorithms.

## 5. Conclusions

Optimal AV delays are patient-specific and differ from nominal device settings. Offset between sensed- and paced AV delay using our proposed quick IEGM-based approach shows a good correlation with the echocardiographic-derived offset. In daily clinical practice, this means that, in non-responders, after echocardiographic sensed AV delay optimization, the paced AV delay can easily be calculated and subsequently programmed. Similar IEGM and echocardiography offsets were associated with response to CRT.

## Figures and Tables

**Table 1:** Baseline characteristics.

	Patient Population (n = 328)
**Demographics**	
Male gender, % (n)	64 (210)
Age, years	69 ± 12
BMI, kg/m^2^	27.8 ± 4.7
Weight, kg	84 ± 15
Height, cm	174 ± 9
**Medical history**	
Hypertension, % (n)	43 (142)
Diabetes mellitus, % (n)	26 (84)
Coronary artery disease, % (n)	50 (164)
Myocardial infarction, % (n)	38 (125)
CABG, % (n)	17 (57)
Dilated cardiomyopathy, % (n)	52 (171)
History of atrial fibrillation, % (n)	20 (66)
**Clinical profile**	
Systolic blood pressure, mmHg	119 ± 20
Diastolic blood pressure, mmHg	72 ± 11
NYHA class, % (n)	
I	3 (10)
II	69 (226)
III	27 (89)
IV	1 (3)
**ECG**	
Heart rate, bpm	73 ± 14
PQ duration, ms	186 ± 41
QRS duration, ms	159 ± 20
**Echocardiography**	
LV ejection fraction, %	25 ± 8
LV end diastolic diameter, mm	61 ± 9
LV end-systolic diameter, mm	51 ± 10
LV end diastolic volume, mL	191 ± 78
LV the end-systolic volume, mL	142 ± 67
LA the end-systolic volume, mL	74 ± 26
LA volume index ^†^, mL/m^2^	38 ± 13
Mitral regurgitation *, % (n)	51 (168)
Tricuspid regurgitation *, % (n)	27 (87)
**Implantation**	
Upgrade, % (n)	22 (73)
CRT-P, % (n)	6 (20)
CRT-D, % (n)	94 (308)
Lead position	
Anterolateral	2 (8)
Lateral	12 (38)
Posterolateral	72 (235)
Posterior	11 (36)
Transvenous LV lead, % (n)	91 (298)
**Medication use**	
β-blocker, % (n)	89 (292)
ACE inhibitor, % (n)	66 (217)
ARB, % (n)	27 (117)
Diuretics, % (n)	77 (251)
Statin, % (n)	51 (167)
OAC, % (n)	44 (145)
ASA, % (n)	38 (124)
**Laboratory values**	
NTproBNP (ng/mL)	1267 (509–2410)
Hb (mmol/L)	8.4 (7.9–9.0)
Creatinine (umol/L)	96 (78–119)
eGFR (mL/min/1.73 m^2^)	64 (47–81)

ACE, Angiotensin-converting enzyme inhibitor; ARB, angiotensin receptor blocker; ASA, acetylsalicylic acid; BMI, body mass index; CABG, coronary artery bypass graft; eGFR, estimated glomerular filtration rate; Hb, hemoglobin; LV, left ventricular; NTproBNP, N-terminal prohormone of brain natriuretic peptide; NYHA, New York Heart Association. * Mitral—and tricuspid regurgitation was defined as regurgitation that was at least moderate. † LA volume index is the left atrial volume according to the biplane Simpson method corrected for body surface area.

**Table 2:** Sensed and paced AV delays on IEGM and during echocardiography optimization using the iterative method.

	Patient Population (N = 328)
**IEGM**	
Atrial sensing (ms)	194 ± 50
Atrial pacing (ms)	269 ± 59
Offset IEGM (ms)	75 ± 25
**Echocardiography**	
Atrial sensing (ms)	75 ± 26
Atrial pacing (ms)	148 ± 32
Offset Echo (ms)	73 ± 18

IEGM, intracardiac electrogram.

**Table 3:** Nominal AV settings and offset dispersion in our patient population.

Nominal AV Settings for Different Manufacturers				
	Sensed AV Delay (ms)	Paced AV Delay (ms)	Offset (ms)	*p*-Value *
**Biotronik**	150/120	190/160	40	<0.001
**Boston Scientific**	120	180	60	<0.001
**LivaNova**	125/80	190/145	65	<0.001
**Medtronic**	100	130	30	<0.001
**Abbott**	150	200	50	<0.001
**AV Offset Dispersion in Our Patient Cohort**				
**AV Offset (ms)**		**Population (%)**		
≤20		0.6		
21–40		3.0		
41–60		28.7		
61–80		46.6		
81–100		18.6		
101–120		1.5		
121–140		0.3		
>140		0.6		

AV, atrioventricular. * *p*-value is a comparison between manufacturer offset and the offset in our patient cohort.

## Data Availability

The data underlying this article will be shared on reasonable request to the corresponding author.

## Supplementary Materials

The following supporting information can be downloaded at: https://www.mdpi.com/article/10.3390/jcm12124138/s1, Figure S1: Flowchart patient selection; Figure S2: Transmitral flow profiles during AV-sensing and -pacing; Figure S3: IEGMs of three major manufacturers.
