# Supplementary material for: Cardiac Resynchronization Therapy beyond Nominal Settings: An IEGM-Based Approach for Paced and Sensed Atrioventricular Delay Offset Optimization in Daily Clinical Practice
Source: J Clin Med. 2023 Jun 20;12(12):4138. doi: 10.3390/jcm12124138 (PMC10299691; doi:10.3390/jcm12124138)
Supplement: Supplementary file 1 [file jcm-12-04138-s001.zip › jcm-2392548-supplementary.pdf]

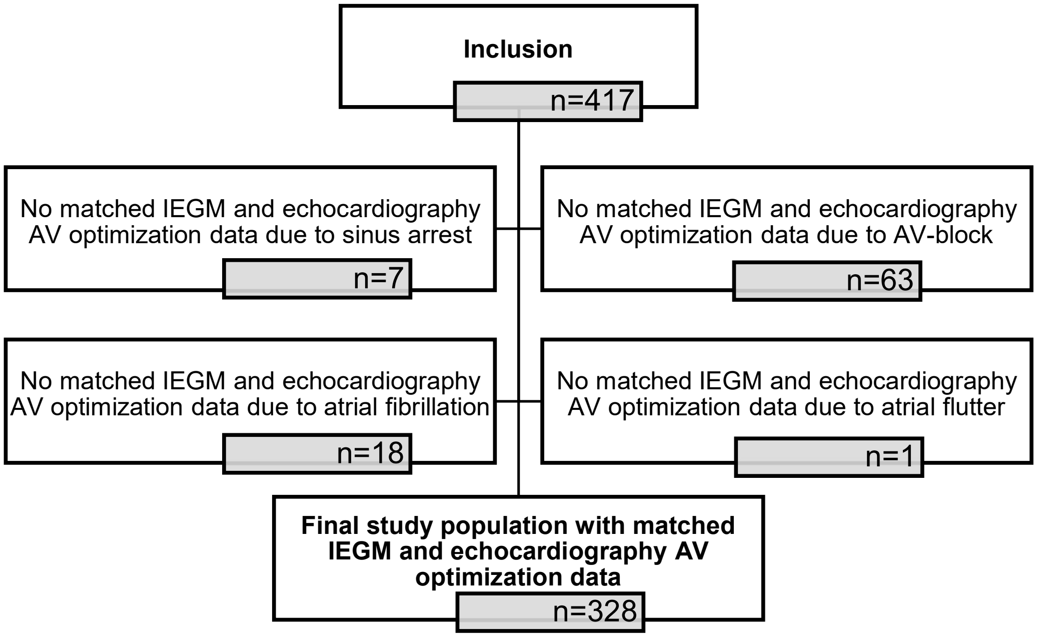

Figure S1. Flowchart patient selection.

Included patient population consisted of 417 patients. For 328 patients matched IEGM and echocardiography data from AV optimization were present, forming the final study population. Reasons for missing data are given in the figure.

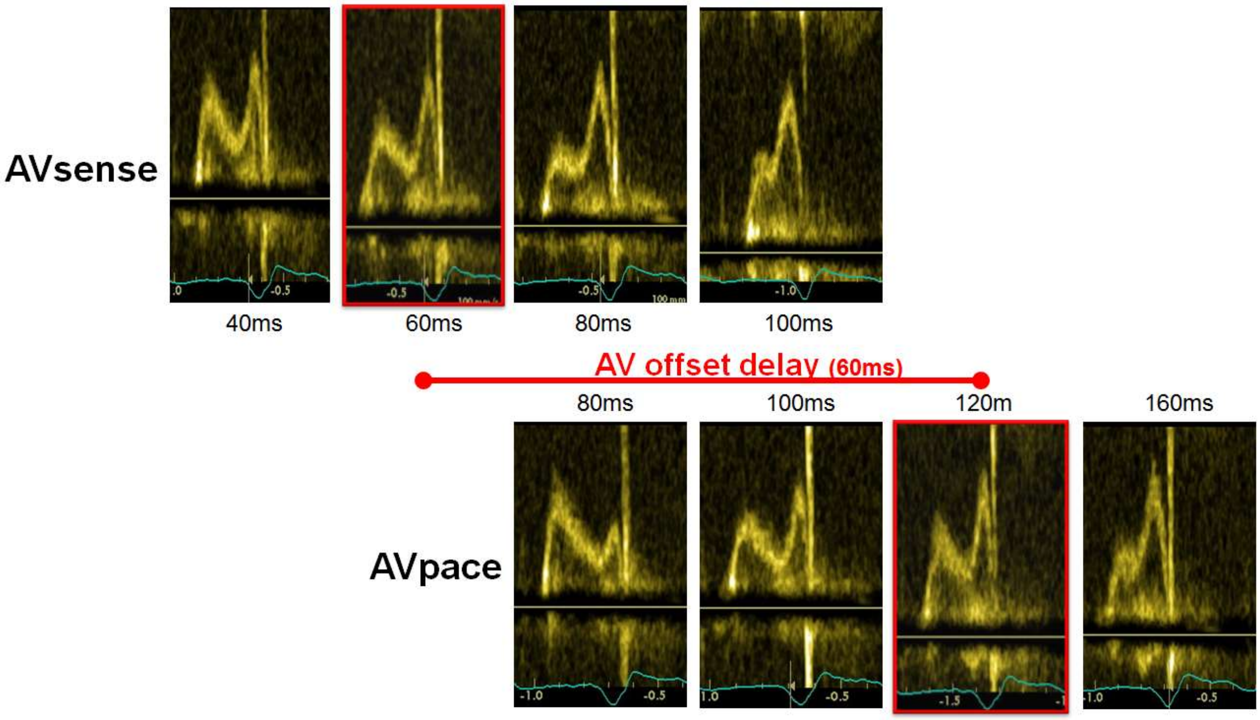

Figure S2. Transmitral flow profiles during AV sensing and -pacing.

During intrinsic sinus rhythm (AVsense) there is A wave truncation at a delay of 40 ms and fusion of the E and A waves on mitral inflow at a delay 80ms, which becomes even more apparent at a delay of 100 ms. Similar transmitral flow profiles can be seen during atrial pacing, albeit at different delays. During pacing (Avpace) A wave truncation is present at a delay of 80ms and 100 ms, while fusion is clearly visible at a delay of 160 ms. For this specific patient optimal sensed AV delay was programmed at 60 ms and optimal paced AV delay was programmed at 120 ms, leading to an AV offset delay of 60 ms.

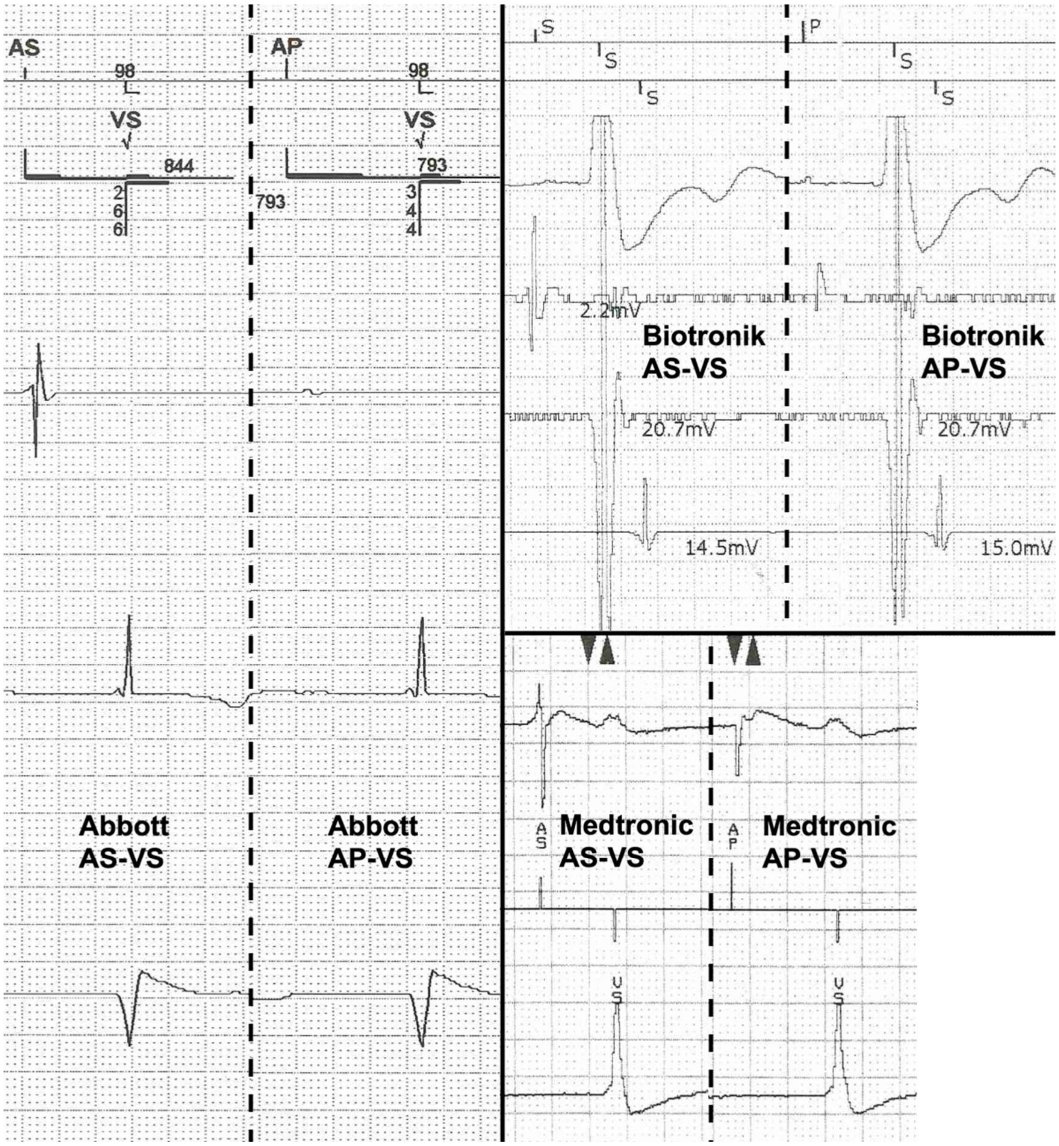

Figure S3. IEGMs of three major manufacturers.

Examples of one atrial and ventricular complex on the IEGM, demonstrating the difference between three manufacturers: Abbott, Biotronik and Medtronic. AS.= atrial sensing. VS = ventricular sensing. AP = atrial pacing.
